# Integrated Transcriptomics and Metabolomics Analysis Reveals the Effects of Cutting on the Synthesis of Flavonoids and Saponins in Chinese Herbal Medicine *Astragalus mongholious*

**DOI:** 10.3390/metabo14020097

**Published:** 2024-01-30

**Authors:** Xu Guo, Xiang Yan, Yuanyuan Wang, Zhiyong Shi, Jingping Niu, Jianping Liang, Xiaoyun Jia

**Affiliations:** 1 College of Life Sciences, Shanxi Agricultural University, Jinzhong 030801, China; gx1196297960@163.com (X.G.); yanx0715@163.com (X.Y.); w13327549040@163.com (Y.W.); shizy@sxau.edu.cn (Z.S.); niujingping@sxau.edu.cn (J.N.); gssjxy@hotmail.com (X.J.); 2 Shanxi Key Laboratory of Chinese Veterinary Medicine Modernization, Shanxi Agricultural University, Jinzhong 030801, China

**Keywords:** *Astragalus mongholicus*, cutting, secondary metabolites, transcriptomics, metabolomics

## Abstract

Astragali Radix, derived from the roots of *Astragalus mongholicus*, is a traditional Chinese medicine containing flavonoids and saponins as its key ingredients. With a shortage in the wild sources of the herbal plant, it is especially important to explore a cultivation mode for *A. mongholicus* for medicinal purposes. Cutting, a physical environmental stress method, was used in this study with the objective of improving the quality of this herbal legume. We found that cutting of the top 1/3 of the aboveground part of *A. mongholicus* during the fruiting period resulted in a significant increase in the content of flavonoids and saponins, as well as in root growth, including length, diameter, and dry weight. Furthermore, the leaves were sampled and analyzed using a combined transcriptome and metabolome analysis approach at five different time points after the treatment. Sixteen differentially expressed unigenes (DEGs) involved in the biosynthesis of flavonoids were identified; these were found to stimulate the synthesis of flavonoids such as formononetin and calycosin–7–O–β–D–glucoside. Moreover, we identified 10 DEGs that were associated with the biosynthesis of saponins, including astragaloside IV and soyasaponin I, and found that they only regulated the mevalonic acid (MVA) pathway. These findings provide new insights into cultivating high-quality *A. mongholicus*, which could potentially alleviate the scarcity of this valuable medicinal plant.

## 1. Introduction

The Chinese herbal medicine Astragali Radix is derived from the dried root of *A. mongholicus* (*Astragalus membranaceus* (Fisch.) Bge. var. *mongholicus* (Bge.) Hsiao), which belongs to the leguminous family [1]. Astragali Radix has been listed in the homology of medicine and food by the National Health Commission of the People’s Republic of China [2]. It has high medicinal value because of its abilities in tonifying qi, lifting yang, and solidifying the surface; it additionally acts as an antiperspirant [3]. Shanxi Province in China is the main origin of *A. mongholicus* [4]. In the 1980s, there was a serious shortage of wild *A. mongholicus* [5]. Therefore, exploring cultivation approaches for *A. mongholicus* is crucial in ensuring better quality of the plant.

The growth and development of plants are often hindered by various stresses, such as physical, chemical, and biological factors, making their living environment unsuitable. The ability of plants to withstand these stresses is known stress resistance [6]. Generally, plants employ metabolic reactions to mitigate, prevent, or repair damage caused by environmental stresses, allowing them to maintain normal physiological activities. However, prolonged and severe stresses that exceed the plant’s compensatory capacity can ultimately result in plant death [7]. With the deepening of research in this field, findings have shown that plants have developed sophisticated protective mechanisms that induce modifications in gene expression when exposed to biological and abiotic infections [8,9]. These changes in gene expression alter their physiology and metabolism [10,11]; these synthesize and accumulate a series of low-molecular-weight compounds that are collectively referred to as phytoalexins. Phytoalexins are usually secondary metabolites which are often the active ingredients of traditional Chinese medicines [7]. Therefore, these environmental stress factors may induce the production and accumulation of medicinal components of traditional Chinese medicine.

Mechanical damage is a prominent physical environmental stress factor; it causes physical harm to plants without affecting their normal physiological activities. This stress factor has been gradually applied in the field of plant production and secondary metabolites [12,13,14,15]. Studies have shown that mechanical damage can stimulate the production of flavonoids, phenylpropanoids, triterpenoids, and amino acid derivatives in plants [16,17,18]. Guo et al. [19] found that cutting the tips of dominant roots had a positive effect on the yield and quality of tobacco, while Lu et al. [20] demonstrated that cutting the base of the trunk of *Ginkgo biloba* can revitalize the plant, leading to an increase in the biomass and flavonoid content of its leaves. Therefore, it is hypothesized that subjecting *A. mongholicus* to appropriate cutting stress might enhance the synthesis of secondary metabolites, such as flavonoids and saponins. 

Flavonoids belong to a group of phenolic compounds and are the main pharmacodynamic components of *A. mongholicus*, which includes flavones, dihydroflavone, dihydrochalcone, isoflavones, flavonols, flavanols, flavanones, and flavonoid glycosides [21]. In plants, phosphoenolpyruvate (PEP) and erythritos–4–phosphate (E4P) are produced through the glycolysis and pentose phosphate pathways [22]. Then, they are converted to shikimic acid and the phenylalanine skeleton via the enzymatic reactions in the shikimic acid pathway [23]. Finally, a variety of flavonoids are synthesized via the phenylpropane pathway [24]. In recent years, flavonoid biosynthesis genes, expressed by many plants, have been extensively studied; these have included *Arabidopsis thaliana* [25], *Camellia sinensis* [26], and *Carthamus tinctorius* L. [27]. Specifically, the transcriptional regulatory mechanism of flavonoids during fruit development [28] and the response mechanism under drought [29] and salt–alkali stress [30] in *A. mongholicus* have been studied. However, the effect of cutting on the content of flavonoids in this species is yet to be elucidated.

Saponins are another important pharmacodynamic component in *A. mongholicus*; these components are mainly triterpenoids that are derived from the pathways that lead to isoprenoid synthesis in *A. mongholicus*. Their precursor, 2, 3–oxidosqualene (OS), is synthesized via the MVA pathway in the cytoplasm and the methyl erythritol phosphate (MEP) pathway in plastids. Oxidative squalene cyclase (OSC) catalyzes the conversion of OS into different sterols and triterpenes; this is a key step in the formation of diverse saponins [31]. OSCs include cyclic artenol synthetase (CAS), beta–amyrin synthetase (*β*–AS), and lupeol synthetase (LUS), which catalyze the synthesis of precursor substances that form saponins in *A. mongholicus* [32,33]. However, the total content of saponins in *A. mongholicus* is low. The content of astragaloside IV in *A. mongholicus* is the highest, but this is still low at only 0.115% [34]. Some studies have shown that abiotic stress factors such as methyl jasmonate [35], ultraviolet light [36], and moisture content [37] can affect the accumulation of saponins of *A. mongholicus* by regulating related genes in the process of saponin synthesis. However, the effect of cutting on the content of saponins in *A. mongholicus* is unknown.

The integrated analysis of transcriptomics and metabolomics is of immense significance in analyzing the internal changes in plants and can provide a sufficient understanding of the molecular mechanisms of plant responses to stresses. Therefore, based on our previous study, we cut 1/3 of the aboveground part of *A. mongholicus* at the fruiting stage to apply stress and performed transcriptomics and metabolomics analyses [38]. The present study aimed to determine an innovative technique for the ecological cultivation of high-quality *A. mongholicus*, helping to alleviate the shortage of *A. mongholicus*.

## 2. Materials and Methods

### 2.1. Plant Materials

In this study, two-year-old *A. mongholicus* with similar growth vigor levels were utilized. The samples were collected from Hunyuan County in Shanxi Province, China (39°52′10.22′′ N, 113°64′98.42′′ E), known for producing genuine *A. mongholicus*. The cutting procedures were carried out at 1/3 of the distance from the top of the stems in the fruiting stage, and a second round of cutting was performed in the subsequent year based on the first year. The samples were collected at fructescence, the deciduous prophase, and the deciduous stage in each year to measure the length, diameter, and dry weight of the roots, as well as the content of flavonoids and saponins (in triplicate), respectively. Control samples, which were left untreated, were also collected during each period of the year.

In addition, fresh leaves below the incision were collected in time and labeled at various time points after cutting in the second year, including 0 h, 4 h, 8 h, 18 h, and 24 h, with six replicates for each time point. They were quickly frozen in liquid nitrogen and stored at −80 °C for transcriptomics and metabolomics analysis.

In this experiment, a total of 66 of *A. mongholicus* plants were collected, with 36 plants utilized to measure the length, diameter, and dry weight of the roots and 30 plants used for transcriptomics and metabolomics analysis of the leaves.

### 2.2. Measurement of Biomass

The length and diameter of the roots were measured using a ruler and a vernier caliper, respectively. Then, they were fixated at 105 °C, dried at 70 °C to obtain a constant weight, and weighed. The measured values were statistically analyzed using ANOVA (*p* < 0.05) with SPSS to detect the significant difference between groups.

### 2.3. Measuring the Contents of Flavonoids and Saponins

The content of total flavonoids was determined using an ultraviolet visible spectrophotometer with rutin as the reference. Two grams of the roots were dried at 60 °C and placed in a 50 mL volumetric bottle to which 35 mL methanol was added and the roots were soaked for 2 h. Then, ultrasonic extraction was performed for 30 min (two times), after which methanol was added to the scale and the mixture was stored for 24 h to obtain the extract of the total flavonoids. Thereafter, 2.0 mL of the extract was added to a 25 mL volumetric flask and mixed with 1.0 mL of 5% sodium nitrite and left to stand for six minutes at room temperature, and then 1.0 mL 10% aluminum nitrite was added and the flask was shaken well. After another 6 min, the samples were mixed with 10.0 mL of 4% sodium hydroxide, water was added to the scale, and the flask was shaken well. After standing for 15 min at room temperature, the absorption value was measured at 500 nm to calculate the content of total flavonoids [39,40,41].

The content of total saponins was determined by vanillin–glacial acetic acid colorimetry with astragaloside IV as the reference. Three grams of the roots were weighed and soaked in 60 mL of methanol in a Soxhlet extractor for 12 h. Then, the mixture was placed in a water bath for 6 h and methanol was recovered. The residue was heated and dissolved with 10 mL water, and then transferred to a liquid separation funnel, to which 30 mL of water was used to saturate n–butanol extraction 4 times. The n–butanol layer was finally obtained and washed 3 times with 20 mL of an ammonia test solution (400 mL concentrated ammonia water mixed with water to obtain a final volume of 1000 mL). Then, the alkali water layer was discarded to recover n–butanol and residue was filled with methanol to 5 mL to obtain the total saponin extraction solution. We determined the absorption value of 0.5 mL of the extract at 540 nm by using the ultraviolet visible spectrophotometer. The content of total saponins was determined according to a method described previously [41].

The measured values were statistically analyzed using ANOVA (*p* < 0.05) with SPSS to detect the significant difference between groups.

### 2.4. Sequencing Data Analysis

Total RNA was extracted using the Trizol reagent (Invitrogen, Carlsbad, CA, USA) by following the manufacturer’s procedure. The total RNA quantity and purity were analyzed using the Bioanalyzer 2100 instrument (Agilent, Santa Clara, CA, USA) and RNA 1000 Nano LabChip Kit (ThermoFisher, Waltham, MA, USA) and the RIN number was > 7.0. Poly(A) RNA was purified in two rounds from total RNA (5 µg) using poly-T oligo-attached magnetic beads. Thereafter, the mRNA was fragmented into small pieces using divalent cations at high temperature. Then, the cleaved RNA fragments were reverse-transcribed to create the final cDNA library in accordance with the protocol provided with the mRNASeq sample preparation kit (Illumina, San Diego, CA, USA). The average insert size for the paired-end libraries was 300 bp (±50 bp). Then, we performed paired-end sequencing using Illumina Novaseq 6000 (LC Sciences, San Diego, CA, USA) following the vendor’s recommended protocol.

First, Cutadapt [42] and perl scripts in house were used to remove the reads that contained adaptor contamination, low-quality bases, and undetermined bases. Then, sequence quality was verified using FastQC (http://www.bioinformatics.babraham.ac.uk/projects/fastqc/, accessed on 3 August 2022), including the Q20, Q30, and GC content of the clean data. All downstream analyses were based on high-quality clean data. De novo assembly of the transcriptome was performed using Trinity 2.4.0 [43]. Trinity transcripts were grouped into clusters based on shared sequence content and these transcript clusters were very loosely referred to as a “gene”. The longest transcript in the cluster was chosen as the “gene” sequence (also known as the unigene). All assembled unigenes were aligned against the non-redundant (Nr) protein database (http://www.ncbi.nlm.nih.gov/, accessed on 11 August 2022), Gene ontology (GO) (http://www.geneontology.org, accessed on 11 August 2022), Kyoto Encyclopedia of Genes and Genomes (KEGG) (http://www.genome.jp/kegg/, accessed on 11 August 2022), and eggNOG (http://eggnogdb.embl.de/, accessed on 11 August 2022) databases using DIAMOND [44] with a threshold of e-value < 0.00001. Salmon [45] was used to determine the expression level of unigenes by calculating TPM [46]. The DEGs were selected with log2 (fold change) ≥ 1 or log2 (FC) ≤ −1 and with statistical significance (*p*–value < 0.05) by using R package edgeR [47]. 

### 2.5. Quantitative Real-Time PCR Analysis

The DEGs associated with flavonoid and terpenoid synthesis were selected for qRT–PCR analysis, which included *PAL*, *C4H*, *FLS*, *COMT*, *β*–*AS*, and *HMGS.* qRT–PCR primers details are provided in Table S1. qRT–PCR was performed using the PerfectStart^®^ Green qPCR SuperMix kit following the manufacturer’s instructions (TransGen Biotech Company, Beijing, China) and the real-time PCR system (StepOnePlus). *Am18S rRNA* was used as the internal reference. The relative expression was determined using the 2^−∆∆CT^ method [48].

### 2.6. Metabolome Analysis 

Leaves of *A. mongholicus* (100 mg) were individually ground in liquid nitrogen and the homogenate was resuspended with prechilled 80% methanol by well vortex. The samples were incubated on ice for 5 min and then centrifuged at 15,000× *g*, 4 °C for 20 min. The supernatant was diluted by adding LC–MS-grade water to obtain a final solution containing 53% methanol. The samples were subsequently transferred to a fresh Eppendorf tube and then centrifuged at 15,000× *g*, 4 °C for 20 min. Finally, the supernatant was injected into the LC–MS/MS system [49]. 

UHPLC–MS/MS analyses were performed using a Vanquish UHPLC system (Thermo Fisher, Bremen, Germary) coupled with an Orbitrap Q Exactive^TM^ HF–X mass spectrometer (Thermo Fisher, Bremen, Germary) in LCSW (Hangzhou, China). Samples were injected into a Hypesil Gold column (100 × 2.1 mm, 1.9 μm) with a 12 min linear gradient at a flow rate of 0.2 mL/min. The eluents for the positive polarity mode were eluent A (0.1% Formic Acid) and eluent B (methanol). The eluents for the negative polarity mode were eluent A (5 mM ammonium acetate, pH 9.0) and eluent B (methanol). The solvent gradient was set as follows: 2% B, 1.5 min; 2–85% B, 3 min; 85–100% B, 10 min; 100–2% B, 10.1 min; 2% B, 12 min. The Q Exactive^TM^ HF–X mass spectrometer was operated in the positive/negative polarity mode with a spray voltage of 3.5 kV, capillary temperature of 320 °C, sheath gas flow rate of 35 psi, an aux gas flow rate of 10 L/min, S–lens RF level of 60, and aux gas heater temperature of 350 °C.

The raw data files generated by UHPLC–MS/MS were processed using Compound Discoverer 3.1 to perform peak alignment, peak picking, and quantitation for each metabolite. The main parameters were set as follows: retention time tolerance, 0.2 min; actual mass tolerance, 5 ppm; signal intensity tolerance, 30%; signal/noise ratio, 3; etc. Thereafter, peak intensities were normalized to the total spectral intensity. The normalized data were used to predict the molecular formula based on additive ions, molecular ion peaks, and fragment ions. Then, the peaks were matched with the mzCloud (https://www.Mzcloud.org/, accessed on 28 August 2022), mzVault, and MassList databases to obtain the accurate qualitative and relative quantitative results. 

The metabolites were annotated using the KEGG (https://www.genome.jp/kegg/pathway.html, accessed on 2 September 2022), HMDB (https://hmdb.ca/metabolites, accessed on 2 September 2022), and LIPIDMaps databases (http://www.lipidmaps.org/, accessed on 2 September 2022). Principal component analysis (PCA) was performed using metaX 1.4.19 (a flexible and comprehensive software for processing metabolomics data). We applied univariate analysis (*t*-test) to calculate the statistical significance (*p*-value). The metabolites with a VIP > 1 and *p*-value < 0.05 and log2 (FC) ≥ 1 or log2 (FC) ≤ −1 were considered to be differential metabolites.

## 3. Results

### 3.1. Effects of Cutting on the Biomass of A. mongholicus

**Figure 1:**
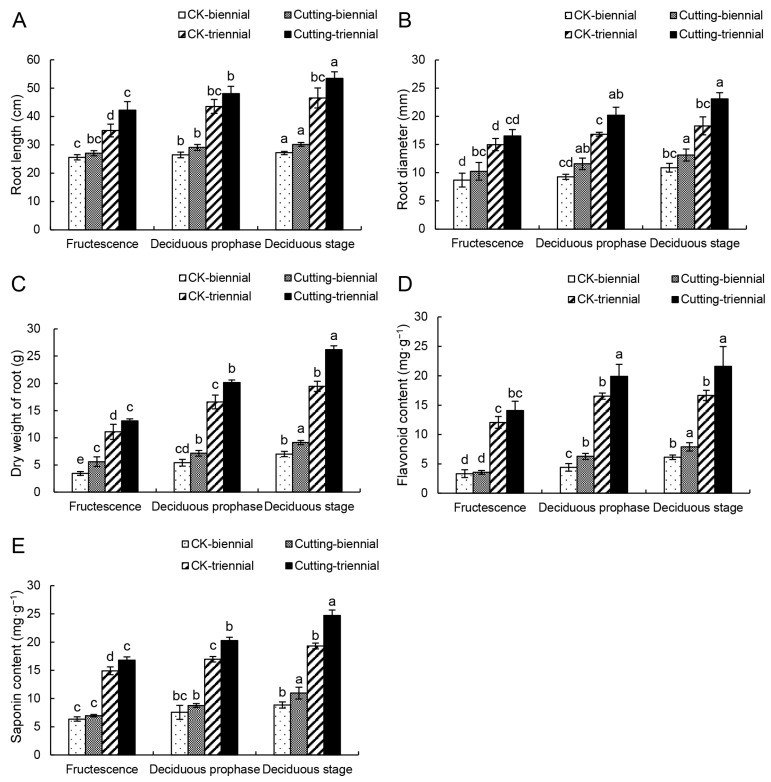
Changes in the biomass and content of flavonoids and saponins. (**A**–**C**) show the effects of cutting on biomass of *A. mongholicus.* (**D**,**E**) show the effects of cutting on the flavonoids and saponins of *A. mongholicus*. Different letters indicate significant difference at 0.05 and biennial and triennial plants were analyzed separately.

Cutting the top 1/3 of the aboveground part of *A. mongholicus* was performed during the fruiting period and these plants as well as control plants were collected at fructescence, the deciduous prophase, and the deciduous stage in each year to measure the biomass (length, diameter, and dry weight) of the roots of *A. mongholicus*. It could be seen that the biomass of the roots of *A. mongholicus* increased significantly after cutting (*p* < 0.05). The effect is more obvious after the second cutting. The root length, diameter, and dry weight of the biennial *A. mongholicus* were increased by 10.7%, 20.8%, and 30.1% compared with those without cutting at the deciduous stage, respectively. The same parameters increased in triennial *A. mongholicus* by 14.9%, 26.2%, and 34.5%, respectively (Figure 1A–C).

### 3.2. Effects of the Cutting on the Content of Total Flavonoids and Saponins 

The roots of *A. mongholicus* were crushed to measure the total flavonoid and saponin content after measuring the biomass. As shown in Figure 1D and E, the flavonoid and saponin content in the roots of *A. mongholicus* were consistent with the changing trend of biomass. The effect was also more pronounced after the second cutting. The content of flavonoids and saponins of the biennial *A. mongholicus* increased by 24.1% and 28%, respectively, and the triennial *A. mongholicus* increased by 28.9% and 29.7%, respectively.

### 3.3. Summary of Transcriptome Analysis after Cutting

The leaves of *A. mongholicus* were collected at five treatment time intervals (0 h, 4 h, 8 h, 18 h, and 24 h) to determine the overall expression of DEGs. The clean data were processed to the final effective fragment. The Q20 of the effective fragments was >97%, the Q30 was >93%, and the GC content was >42% (Table S2). These fragments were reassembled to produce a total of 97,144 unigenes (Table S3). The length distribution and the GC content of the assembly results are presented in Figures S1 and S2. These results indicate that sequencing data were available in sufficient quantity and quality to ensure accurate sequence assembly and adequate transcriptome coverage. The Pearson coefficient of each sample that underwent the same treatment was between 0.55 and 0.90, indicating good duplication among samples (Figure S3).

To determine the corresponding function of unigenes, annotation was carried out in the following six databases: GO, KEGG, Pfam, swissprot, eggNOG, and NR. A total of 97144 unigenes were annotated at a comment rate of 100%. The number of notes was 41549 (45.29%), 28878 (31.48%), 38106 (41.54%), 32067 (34.95%), 49642 (54.11%), and 46967 (51.19%) (Table S4).

GO analysis indicated that the term “biological process” and “regulation of transcription, DNA–templated” were prominent in the “biological process” ontology, “nucleus” and “cytoplasm” in the “cellular component” ontology, and “molecular function” and “protein binding” in the “molecular function” ontology (Figure S4). KEGG analysis showed that 901 and 540 unigenes were related to the “biosynthesis of other secondary metabolites” and “metabolism of terpenoids and polyketides”, respectively (Figure S5). These annotation results provide valuable information for the analysis of the metabolic process of *A. mongholicus*.

### 3.4. DEGs Analysis of A. mongholicus after Cutting

**Figure 2:**
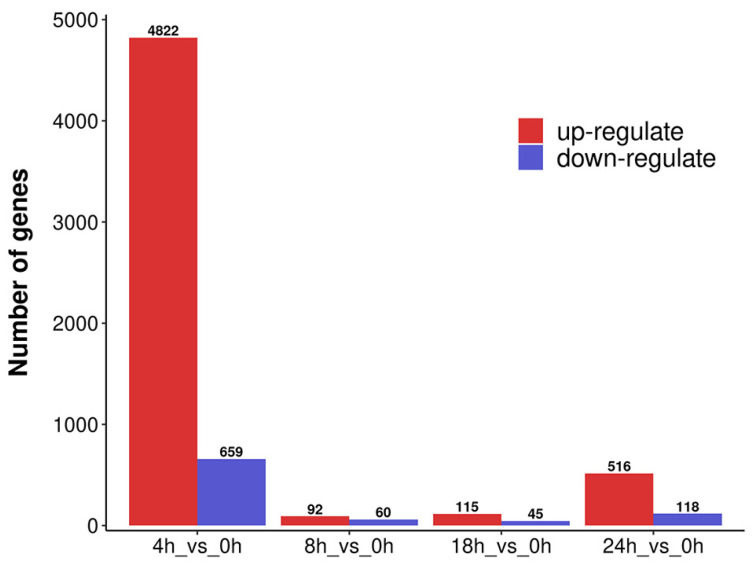
Summary statistics of DEGs.

Based on the TPM value of unigene expression, DEGs of four comparison groups were obtained (Figure 2). The selection criteria were |log 2 (FC)| ≥ 1 and *p*-value < 0.05. We identified 5481 (4822 upregulated and 659 downregulated), 152 (92 upregulated and 60 downregulated), 160 (115 upregulated and 45 downregulated), and 634 (516 upregulated and 118 downregulated) DEGs from the 4 h vs. 0 h, 8 h vs. 0 h, 18 h vs. 0 h, and 24 h vs. 0 h comparison groups, respectively.

**Figure 3:**
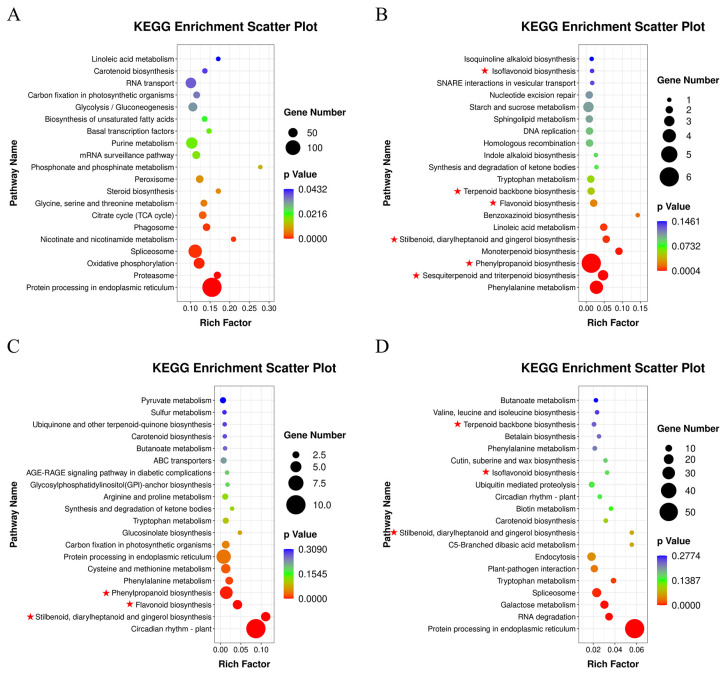
KEGG enrichment of DEGs in the four groups. The degree of enrichment was measured by a rich factor, *p*-value, and the number of unigenes enriched to each KEGG term. Rich factor refers to the ratio of the number of DEGs enriched to the number of all unigenes annotated in each term. The greater the rich factor, the greater the enrichment. The red star signs indicate the pathways related to the synthesis of terpenoids and flavonoids. (**A**): DEG enrichment terms in 4 h vs. 0 h; (**B**): DEG enrichment terms in 8 h vs. 0 h; (**C**): DEG enrichment terms in 18 h vs. 0 h; (**D**): DEG enrichment terms in 24 h vs. 0 h.

The top 20 enrichment pathways of KEGG in the four comparison groups are shown in Figure 3. The most enrichment pathways of the DEGs were recorded at 4 h vs. 0 h, and the upregulated expression unigenes accounted for 94.23% (736/781) (Table S5). These results indicate that 0–4 h was the strongest period of response to cutting. In 4 h vs. 0 h, the most DEGs were concentrated in “Protein processing in endoplasmic reticulum” (ko04141), “Spliceosome” (ko03040), “RNA transport” (ko03015), and other pathways related to the expression of genetic information, followed by “Glycolysis/Gluconeogenesis” (ko00010), “Citrate cycle” (ko00020), and other pathways related to carbohydrate (Figure 3A). The two comparison groups of 8 h vs. 0 h and 18 h vs. 0 h showed great similarities in the significantly different pathways, including “Phenylpropanoid biosynthesis” (ko00940), “Flavonoid biosynthesis” (ko00941), and “Stilbenoid, diarylheptanoid and gingerol biosynthesis" (ko00945), which were related to flavonoid synthesis. "Terpenoid backbone biosynthesis” (ko00900) and “Sesquiterpenoid and triterpenoid biosynthesis” (ko00909) related to terpenoid synthesis were only significantly enriched in the 8 h vs. 0 h comparison (Figure 3B,C). In 24 h vs. 0 h, the DEGs were mainly concentrated in “Protein processing in endoplasmic reticulum” and “Spliceosome” (Figure 3D). Therefore, these DEGs related to the synthesis of flavonoids and terpenoids were highly likely to be responsible for the positive accumulation of the two classes of secondary metabolites.

**Figure 4:**
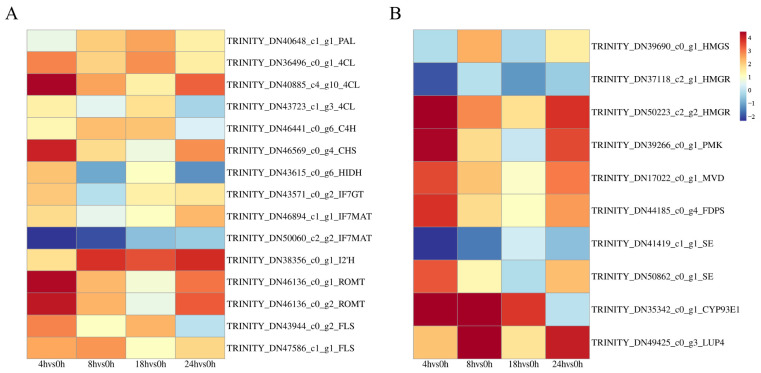
DEG heat map. (**A**): The heat map of DEGs related to flavonoid biosynthesis. (**B**) The heat map of DEGs related to saponin biosynthesis. The four grids from the left to the right represent the Log2 (FC) values of difference multiple of DEGs in 4 h vs. 0 h, 8 h vs. 0 h, 18 h vs. 0 h, and 24 h vs. 0 h.

Fifteen DEGs related to flavonoid synthesis were involved in four pathways (Figure 4A, Table S6). The enzymes encoded by DEGs included phenylalanine ammonia lyase (PAL, 1 unigene), trans–cinnamate 4–monooxygenase (C4H, 1 unigene), and 4–coumarate–CoA ligase (4CL, 3 unigenes) in “Phenylpropanoid biosynthesis”, chalcone synthase (CHS, 1 unigene) and flavonol synthase (FLS, 2 unigenes) in “Flavonoid biosynthesis”, 2–hydroxyisoflavone dehydratase (HIDI, 1 unigene), isoflavone 2′–hydroxylase (I2′H, 1 unigene), isoflavone 7–O–glucosyltransferase (IF7GT, 1 unigene), and isoflavone 7–O–glucoside-6” –O–malonyl transferase (IF7MAT, 2 unigenes) in “Isoflavonoid biosynthesis”, and pterostilbene synthase (ROMT, 2 unigenes) in “Stilbene, diarylheptane and gingerol biosynthesis”.

*A. mongholicus* also produces a variety of saponins, which are another important secondary metabolite. So far, more than 50 saponins have been isolated from Astragali radix. Therefore, the quality of *A. mongholicus* could be significantly improved by increasing the content of saponins. Ten DEGs associated with saponin synthesis were involved in two pathways. The enzymes encoded by DEGs included hydroxymethyl glutaryl–CoA synthase (HMGS, 1 unigene), hydroxymethyl–glutaryl CoA reductase (HMGR, 1 unigene), phosphomevalonate kinase (PMK, 1 unigene), diphosphomevalonate decarboxylase (MVD, 1 unigene), and farnesyl diphosphate synthase (FDPS, 1 unigene) in “Terpenoid backbone biosynthesis”, and squalene epoxidase (SE, 2 unigenes), *β*–amyrin synthase (*β*–AS, 1 unigene), and *β*–amyrin 24–hydroxylase (CYP93E1, 1 unigene) in “Sesquiterpenoid and triterpenoid biosynthesis” (Figure 4B, Table S7).

### 3.5. Quantitative PCR Validation of DEGs

**Figure 5:**
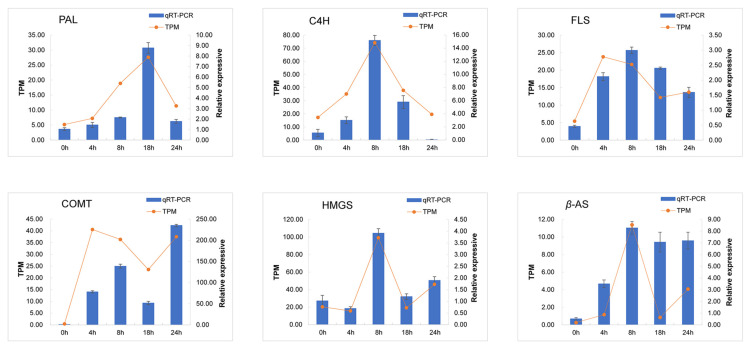
The integrated analysis of qRT–PCR and RNA–Seq validation. The orange line represents the TPM value of the unigenes, and the blue bar represents the qRT–PCR result. The value is expressed as average of three biological replicates.

To further analyze the reliability of the RNA–Seq data, six genes associated with synthesis of flavonoids and saponins were selected for qRT–PCR analysis. The relative changes in candidate gene expression are shown in Figure 5, indicating the reliability of the results.

### 3.6. Metabolome Analysis of A. mongholicus after Cutting

**Figure 6:**
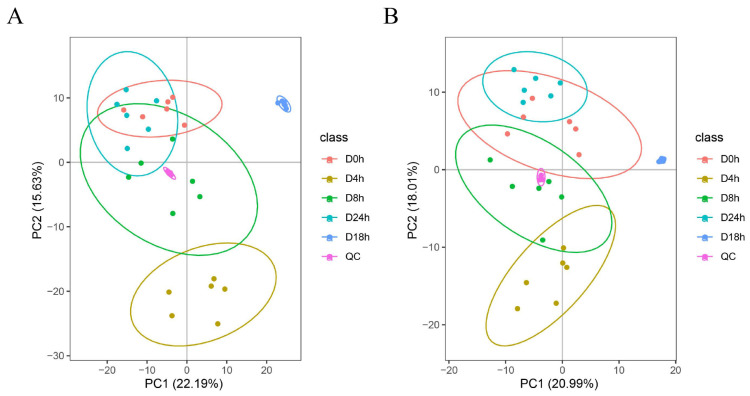
Principal component analysis of metabolites identified. (**A**): Positive ions, (**B**): negative ions.

Before analyzing the differentially accumulated metabolites (DAMs), PCA analysis was performed to determine the degree of variation between and within groups (Figure 6). Both the positive and negative scan modes were able to isolate 30 samples using the first two main components. In the positive-ion mode, PC1 accounted for 22.19% and PC2 accounted for 15.6%, whereas in the negative-ion mode, PC1 and PC2 accounted for 20.99% and 18.01%, respectively. The principal components at 0 h and 24 h were similar. Moreover, QC enrichment of the quality control samples was better.

**Figure 7:**
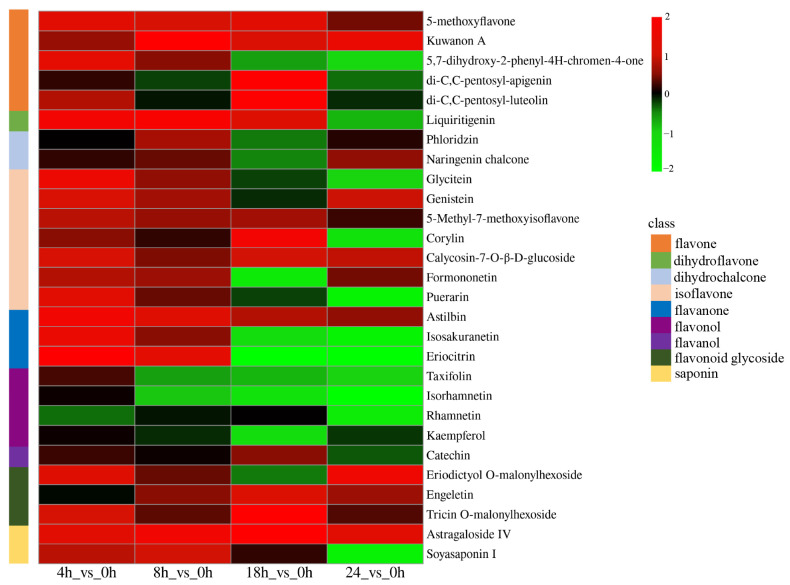
Correlation analysis of the content of flavonoids and saponins. The four grids from the left to the right represent the Log2 (FC) values of difference multiple of DAMs in 4 h vs. 0 h, 8 h vs. 0 h, 18 h vs. 0 h, and 24 h vs. 0 h.

To further explore the DAMs of *A. mongholicus*, the UHPLC–MS/MS system was used to analyze the non-widely targeted metabolome (Figure 7, Table S8). In total, we detected twenty-six differently accumulated flavonoids, including five flavones (5–methoxyflavone, 5,7–dihydroxy–2–phenyl–4H–chromen–4–one, Kuwanon A, di–C,C–pentosyl-apigenin and di–C,C–pentosyl–luteolin), one dihydroflavone (Liquiritigenin), 2 dihydrochalcones (Phloridzin, Naringenin and chalcone), seven isoflavones (Glycitein, Genistein, Puerarin, 5–Methyl–7–methoxyisoflavone, Corylin, Calycosin–7–O–β–D–glucoside and formononetin), three flavanones (Eriocitrin, Astilbin and Isosakuranetin), four flavonols (Kaempferol, Taxifolin, Isorhamnetin and Rhamnetin), one flavanol (Catechin), three flavonoid glycosides (Eriodictyol O–malonylhexoside, Engeletin and Tricin O–malonylhexoside), and two kinds of saponins, including astragaloside IV and Soyasaponin I.

### 3.7. Integrated Analysis of Transcriptomics and Metabolomics

**Figure 8:**
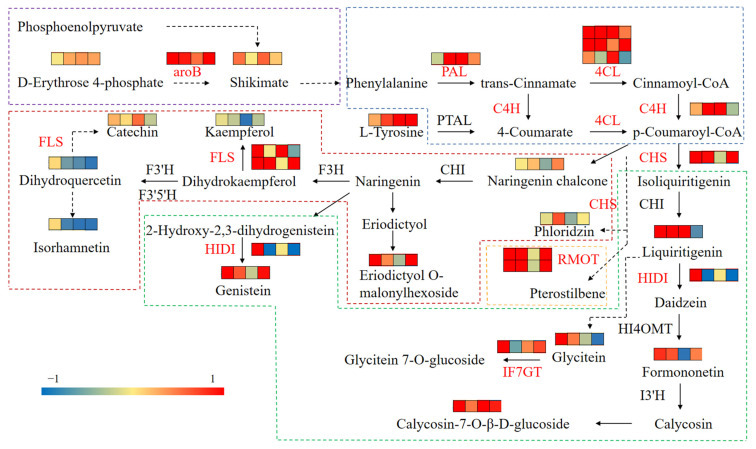
The DEGs and DAMs involved in flavonoid biosynthesis in response to cutting. Dotted lines with multiple arrows represent multiple enzymatic catalyzed steps. The red text indicates that the unigenes related to this enzyme are differentially expressed. The four grids from the left to the right represent the Log2 (FC) values of difference multiple of DEGs or DAMs in 4 h vs. 0 h, 8 h vs. 0 h, 18 h vs. 0 h, and 24 h vs. 0 h, respectively. Phenylalanine, tyrosine, and tryptophan biosynthesis pathways are marked in purple box. Phenylpropanoid biosynthesis pathway is marked in blue box. Flavonoid biosynthesis pathway is marked in red box. Isoflavonoid biosynthesis pathway is marked in green box. Stilbenoid, diarylheptanoid, and gingerol biosynthesis pathways are marked in orange box.

Integrated transcriptomics and metabolomics data showed that cutting affected the flavonoid biosynthesis pathway in *A. mongholicus* (Figure 8). The glycolysis and pentose phosphate pathways produce PEP and E4P, respectively, which were converted to shikimic acid by enzymatic reaction through the shikimic acid pathway, and further converted to phenylalanine via the phenylpropane pathway. In addition, only the regulatory unigene of aroB was significantly upregulated in the “Phenylalanine, tyrosine and tryptophan biosynthesis” pathway (ko00400). L–Tyrosine directly entered the phenylpropane pathway through the conversion of PTAL to 4–Coumarate. The content of E4P and shikimic acid were elevated throughout the cutting stage, and the content of L–tyrosine also showed a significant increase after 8 h. As shown in Figure 8, the unigenes encoding PAL and 4CL were significantly upregulated after cutting. The unigenes encoding C4H were upregulated at first and downregulated at 24 h. These upstream products were further converted to naringenin chalcone, phloridzin, and isoliquiritigenin into the synthesis pathways of flavones, dihydroflavones, dihydrochalcones, isoflavones, flavonols, flavanols, flavanones, and flavonoid glycosides. In the “Flavonoid biosynthesis” pathway, the unigenes associated with CHS and FLS showed an upregulation trend over the entire 24 h, and P–coumaryl CoA was catalyzed by CHS and CHI to produce liquiritigenin, which further synthesizes isoflavones; the content of liquiritigenin was increased 1.26–3.43 times before 24 h. In addition, five DEGs related to isoflavone synthesis were identified, encoding HIDI, IF7GT, IF7MAT, and I2′H, respectively. Among them, the unigenes related to HIDI and IF7GT showed a trend of up–down–up, whereas of the two unigenes related to IF7MAT, one was continuously downregulated and the other was upregulated. Liquiritigenin was catalyzed by HIDI to form daidzein, which was then catalyzed by HI4MOT to form formononetin. Finally, formononetin was catalyzed by I3’H to form calycosin and calycosin–7–O–β–D–glucoside, which increased 1.40–2.15 times. Liquiritigenin was converted to glycitein by another pathway, but the content of glycitein was significantly increased only before 8 h. Naringin chalcone was catalyzed by CHI to produce naringenin, which then produced dihydrokaempferol, 2–hydroxy–2, 3–dihydrogenistein, and eriodictyol into different pathways. However, it is interesting to note that flavonols, including kaempferol, dihydroquercetin, isorhamnetin, and rhamnetin, overall decreased. The level of genistein, an end product, increased 1.62–2.18 times (*p* < 0.05). Although the content of flavonoids such as eriodictyol did not change significantly, most of its products such as eriodictyol O–malonylhexoside increased significantly. In conclusion, the DEGs related to flavonoid synthesis were generally upregulated and the flavonoid content in the leaves of *A. mongholicus* was generally increased.

**Figure 9:**
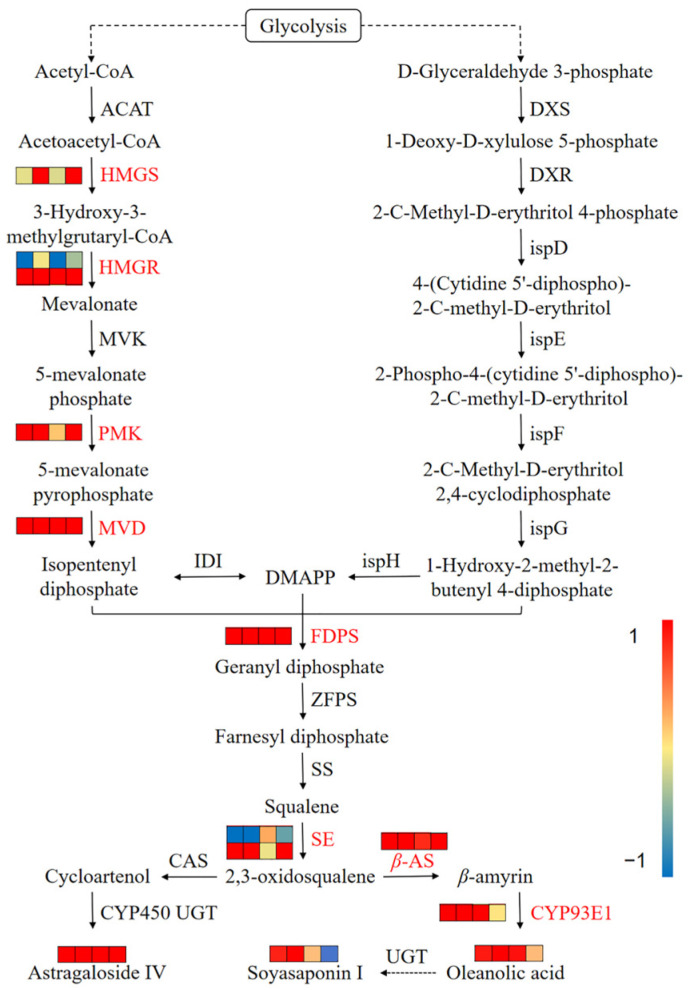
The DEGs or DAMs involved in saponin biosynthesis in response to cutting. The red text indicates that the unigenes related to this enzyme are differentially expressed. The four grids from the left to the right represent the Log2 (FC) values of difference multiple of DEGs or DAMs in 4 h vs. 0 h, 8 h vs. 0 h, 18 h vs. 0 h, and 24 h vs. 0 h, respectively.

Among the two pathways of MVA and MEP for terpenoid synthesis, only some unigenes related to enzymes in the MVA pathway were differentially expressed (Figure 9). Specifically, the unigene encoding HMGS demonstrated an upregulated trend. OS formed *β*–amyrin under the action of *β*–AS, which was then converted into oleanolic acid by CYP93E1. The level of oleanolic acid was increased by 1.12–7.20 times. As a saponin compound converted from oleanolic acid, the content of soyasaponin I increased by 1.13–1.99 times within 18 h of the treatment. Although the *CAS* was unchanged, the content of astragaloside IV was observed to increase by 2.56–4.10 times.

## 4. Discussion

During the growth of plants, the aboveground and underground parts are interdependent. If any of the components are under stress, it results in changes of numerous proteins and secondary metabolites [50,51]. Flower removal has been reported to increase the yield and total biomass of *Helianthus tuberosus* organs [52]. By cutting off the top 5 cm of *Scutellaria baicalensis Georgi*, the yield of its roots, stems, and leaves increased, in addition to the concentration of effective ingredients such as flavonoids and baicalin [53]. The results of the present study indicated that when 1/3 of the aboveground parts of *A. mongholicus* was cut during its fruiting stage, it had a positive effect on the root growth (length, diameter, and dry weight), as well as the accumulation of total flavonoids and saponins (Figure 1).

When plants are exposed to environmental stresses, some unigenes related to stress resistance are activated to facilitate the accumulation of metabolic substances, enabling the plant to withstand and adapt to the stresses [54]. In our study, unigenes related to the expression of genetic information and the carbohydrate synthesis exhibited the strongest response to cutting in the 4 h vs. 0 h group (Figure 2). This suggests that, during the early stages of cutting, *A. mongholicus* underwent extensive internal regulation to maintain its growth, which promoted the synthesis of abundant primary metabolites and gradually induced the synthesis of secondary metabolites such as flavonoids and terpenoids. Subsequently, with the passage of time, the growth of *A. mongholicus* adapted to the stress and the unigenes gradually returned to their normal levels [17]. This was further supported by the principal component analysis (Figure 6). To further explore the molecular mechanism of cutting that promoted flavonoid and saponin production in *A. mongholicus*, we compared the differences in transcriptome and metabolome levels between the treatment and control groups [55,56]. The DEGs related to the synthesis of flavonoids and saponins were identified by KEGG analysis.

There were 16 DEGs related to flavonoid synthesis. In general, “Phenylpropanoid biosynthesis” is a common upstream pathway for the synthesis of flavonoids and other phenolic compounds such as tannins and anthocyanins [57]. PAL is the first key enzyme in phenylpropanoid biosynthesis, and the first defense gene found in plants [58,59]. C4H and 4CL are key enzymes involved in the second step of this pathway, and C4H is considered a rate-limiting enzyme in this pathway [60]. In our study, the expression of five regulatory unigenes of these three enzymes was significantly upregulated. We speculated that cutting induced the upregulation of upstream pathway genes for flavonoid synthesis, thereby accelerating the conversion of upstream compounds. The content of E4P, which is one of the sources of precursor substances and shikimic acid, significantly increased after cutting, which provided a rich basis for flavonoid synthesis. Interestingly, only one unigene related to aroB was upregulated, perhaps because it is the key enzyme in the shikimic acid pathway. CHS is a key rate-limiting enzyme in the “flavonoid biosynthesis” pathway [60]. This might be the reason for the increase in liquiritigenin content, which is a precursor of isoflavones [29]. In the entire process of flavonoid biosynthesis, their diversity was caused by the modification reactions of cytochrome P450 oxidase (CYP450), UDP–glycosyl transferase (UGT), and o–methyltransferase (OMT) [61]. In this study, *CYP73A16*, the regulatory unigene of rate-limiting enzyme C4H, and *CYP81E9*, the regulatory unigene of I′2H, were significantly upregulated. The increase in the content of formononetin was particularly notable in the early stage of treatment, likely due to its conversion into calycosin–7–O–β–D–glucoside, which is a quality control standard of *A. mongholicus* [1]. The final step in flavonoid biosynthesis involves glycoylation catalyzed by UGT, which promotes the solubility, stability, and bioactivity of secondary metabolites in response to environmental changes. Flavonoids can be catalyzed by different UGTs to produce different glycosides [62,63,64,65]. IF7GT is the main UGT in the flavonoid synthesis pathway of *A. mongholicus*. The unigene regulated to IF7GT was also significantly upregulated after cutting and promoted the synthesis of flavonoid glycosides. It might be the reason why the glycitein decreased after 8 h of treatment. Therefore, these unigenes that modified enzymes play a significant role in promoting the synthesis and diversity of flavonoids of *A. mongholicus* after cutting. In other words, we can increase the content of flavonoids by improving the expression of genes related to key enzymes such as aroB, PAL, C4H, FLS, etc. Furthermore, the overall reduction in flavonols may be due to their limited role in the resistance to the stress in *A. mongholicus*, as well as potential internal resource competition for the synthesis of flavones and isoflavones [62].

Saponins belong to the terpenoid family and play an important role in resisting environmental stress [32]. In the present study, we found that cutting induced the differential expression of key enzyme genes in the MVA pathway to promote the synthesis of terpenoids such as saponins [66]. HMGR is a rate-limiting enzyme for terpenoid synthesis [67]. Therefore, we believed that the upregulated expression of HMGR-, PMK-, and MVD-related unigenes promoted the synthesis of isopentenyl pyrophosphate, and the upregulated expression of SE-, *β*–AS-, and CYP93E1-related unigenes further promoted the synthesis of oleanlic acid and soyasaponin I [36,37]. After 24 h of treatment, the expression level of *CYP93E1* returned to the original state, and the content of oleanolic acid also decreased, which affected the content of soyasaponin I. This suggests that CYP93E1 may act as the rate-limiting enzyme for the synthesis of oleane-type saponin in *A. mongholicus*. In addition, the unigenes regulating HMGR and SE expression were both upregulated and downregulated, which might be related to the negative feedback regulation in the process of terpenoid backbone biosynthesis and modification [68]. However, the specific downstream pathway of saponin synthesis remains to be further elucidated.

## 5. Conclusions

Through integrated transcriptomics and metabolomics analysis, we determined the effects of cutting the top 1/3 of the aboveground part of *A. mongholicus* during the fruiting period on the content of flavonoids and saponins. Our results showed that cutting affected the differential expression of unigenes (*aroB*, *PAL*, *C4H*, *4CL*, *CHS*, *IF7GT*, *IF7MAT*, *I2′H*, and *FLS*) related to flavonoid synthesis, which stimulated the increase in the synthesis of flavonoids such as formononetin and calycosin–7–O–β–D–glucoside. Furthermore, cutting induced the differential expression of key enzyme unigenes (*HMGR*, *PMK*, *MVD*, *SE*, *β*–*AS*, and *CYP93E1*) in the MVA pathway to promote the synthesis of astragaloside IV and soyasaponin I. Additionally, the root length, diameter, and dry weight of *A. mongholicus* were also significantly improved within two years of cutting. This research provides a new idea for ecological cultivation technology to enhance the quality and yield of *A. mongholicus* and lays a foundation for improving the quality of *A. mongholicus* through transgenic approaches.

## Data Availability

The data presented in this study are available in the article and Supplementary Material.

## Supplementary Materials

The following supporting information can be downloaded at: https://www.mdpi.com/article/10.3390/metabo14020097/s1. Figure S1: Length Distribution of unigene. Figure S2: GC content in unigenes. Figure S3: Pearson correlation of samples under different processing times. Figure S4: Gene ontology classification of A. mongholicus unigenes. Figure S5: KEGG classification of A. mongholicus unigenes. Table S1: qRT–PCR primers used in this study. Table S2: Summary statistics of sequencing results. Table S3: Summary statistics of unigenes. Table S4: Summary of unigenes function annotation result. Table S5: The enrichment pathways of the DEGs in 4 h vs. 0 h. Table S6: Expression of unigenes related to flavonoid synthesis. Table S7: Expression of unigenes related to saponin synthesis. Table S8: Differential accumulation metabolites.

## Abbreviations

DEGs, differentially expressed genes; DAMs, different accumulated metabolites; PEP, phosphoenolpyruvate; E4P, erythritos–4–phosphate; UHPLC–MS/MS, ultra–high performance liquid chromatography tandem mass spectrometry; PAL, phenylalanine ammonia lyase; C4H, trans–cinnamate 4–monooxygenase; 4CL, 4–coumaroyl CoA ligase; CHS, chalcone synthase; CHI, chalcone isomerase; OS, 2, 3–oxidosqualene; MVA, mevaleric acid; MEP, methyl erythritol phosphate pathway; OSC, Oxidative squalene cyclase; CAS, cyclic artenol synthetase; *β*–AS, beta–amyrin synthetase; LAS, lanosterol synthetase; LUS, lupeol synthetase; BP, biological process, CC, cellular component; MF, molecular function; Nr, non–redundant; GO, Gene ontology; KEGG, Kyoto Encyclopedia of Genes and Genomes; PCA, Principal components analysis; FLS, flavonol synthase; HIDI, 2–hydroxyisoflavone dehydratase; I2′H, isoflavone 2′–hydroxylase; IF7GT, isoflavone 7–O–glucosyltransferase; IF7MAT, 7–O–glucoside–6′′–O–malonyl transferase; ROMT, pterostilbene synthase; ACAT, acetyl–CoA C–acetyltransferase; HMGS, hydroxymethyl glutaryl-CoA synthase; HMGCR, hydroxymethyl–glutaryl CoA reductase; MVK, mevalonate kinase; PMK, phosphomevalonate kinase; MVD, diphosphomevalonate decarboxylase; FDPS, farnesyl diphosphate synthase; ZFPS, farnesyl diphosphate synthase; SS, squalene synthase; SE, squalene epoxidase; CYP450, cytochrome P450 oxidase; UGT, UDP–glycosyl transferase; OMT, o–methyltransferase; F3′H, flavanone 3′–hydroxylase; F3′5′H, flavonoid 3′, 5′-hydroxylase; F3H, naringenin 3–dioxygenase; aroB, 3–dehydroquinate synthase; PTAL, phenylalanine/tyrosine ammonia–lyase.
