# Supplementary material for: Integrated Transcriptomics and Metabolomics Analysis Reveals the Effects of Cutting on the Synthesis of Flavonoids and Saponins in Chinese Herbal Medicine Astragalus mongholious
Source: Metabolites. 2024 Jan 30;14(2):97. doi: 10.3390/metabo14020097 (PMC10891646; doi:10.3390/metabo14020097)
Supplement: Supplementary file 1 [file metabolites-14-00097-s001.zip › Supplementary Files.pdf]

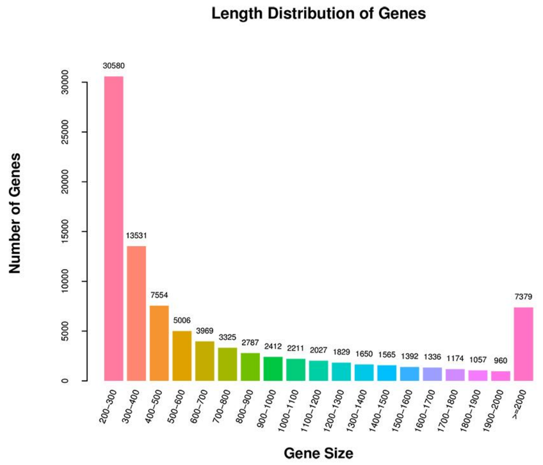

Fig. S1 Length distribution of unigene

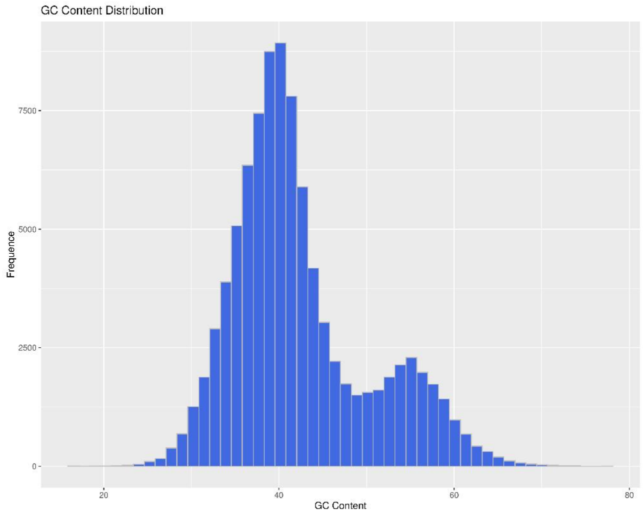

Fig. S2 GC content in unigenes

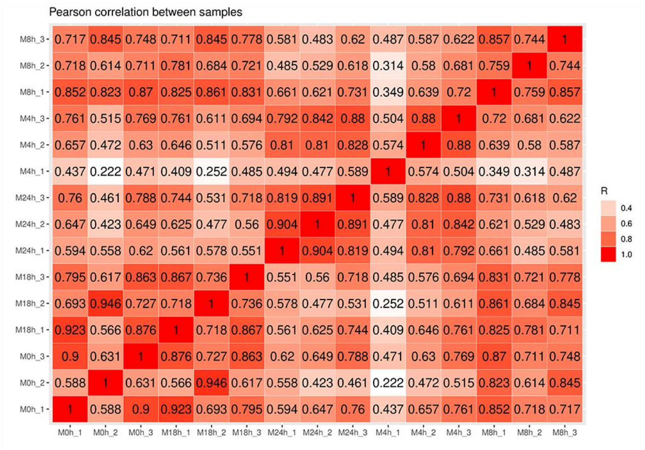

Fig. S3 Pearson correlation of samples under different processing times

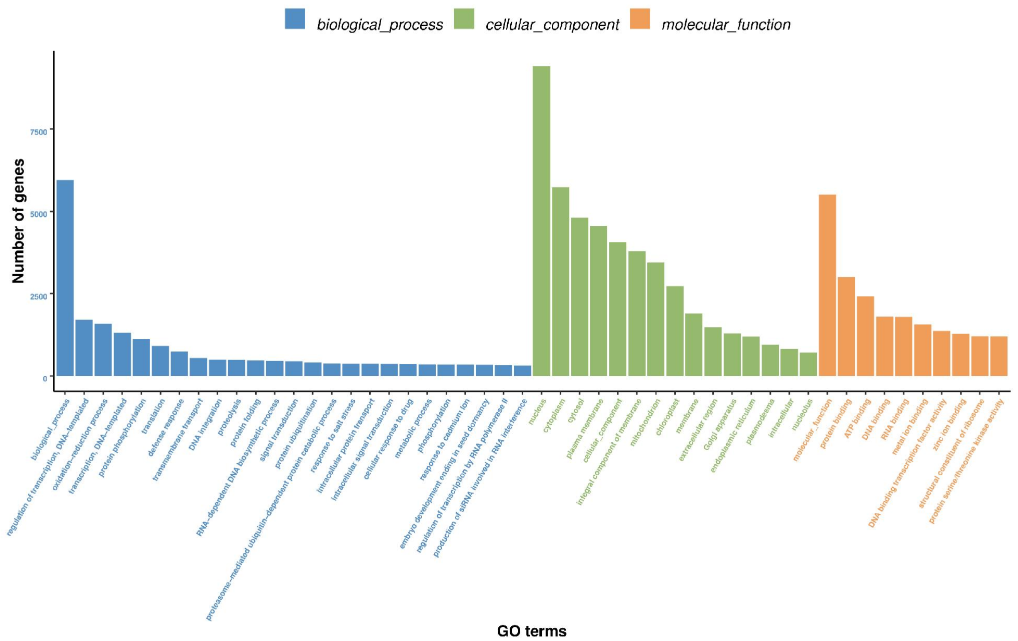

Fig. S4 Gene ontology classification of *A. mongholicus* unigenes

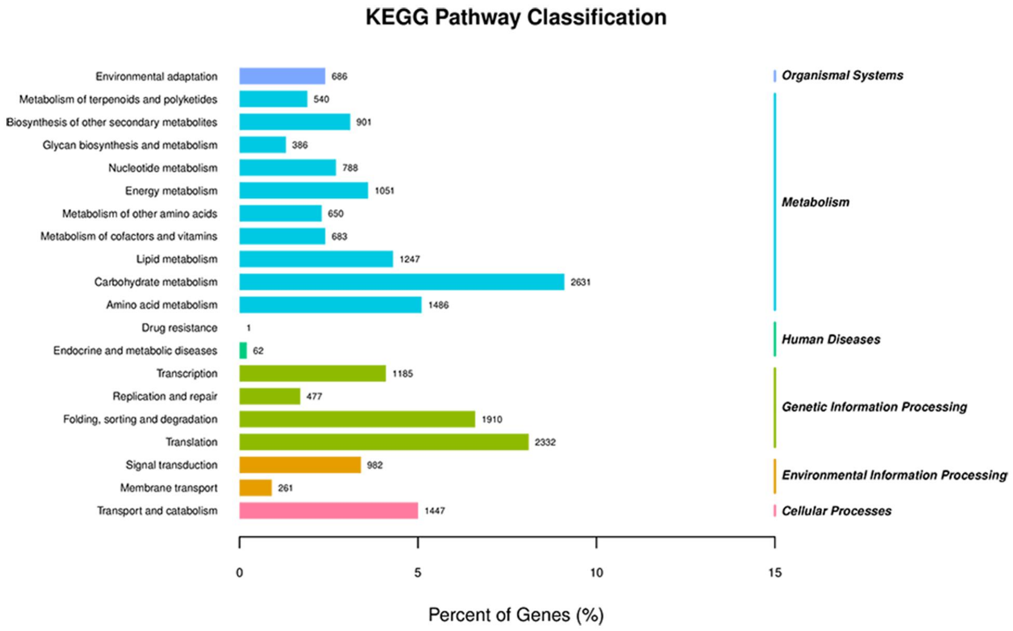

Fig. S5 KEGG classification of *A. mongholicus* unigenes.
